# Controlling Epitaxial Zn Growth via Guided Laser Patterning toward High‐Performance 6Ah Aqueous Zn||I_2_ Batteries

**DOI:** 10.1002/advs.78252

**Published:** 2026-10-15

**Authors:** Suyeon Kim, Dongjae Kim, Youngeun Kim, Chaejeong Kim, Myunghyun Ryu, Wooyoung Jin, Hyeokjin Kwon, Kyu‐Nam Jung, Ki Jae Kim, Jong‐Won Lee, Jinhong Lee

**Affiliations:** ^1^ Renewable Energy Institute Korea Institute of Energy Research (KIER) Daejeon Republic of Korea; ^2^ Department of Future Energy Engineering Sungkyunkwan University Suwon Republic of Korea; ^3^ Division of Materials Science and Engineering Hanyang University Seoul Republic of Korea; ^4^ Ulsan Advanced Energy Technology R&D Center Korea Institute of Energy Research (KIER) Ulsan Republic of Korea; ^5^ Department of Energy Science and Engineering Daegu Gyeongbuk Institute of Science and Technology (DGIST) Daegu Republic of Korea; ^6^ Department of Energy Science Sungkyunkwan University Suwon Republic of Korea

**Keywords:** hydrogen evolution, laser patterning, long‐term cycle, Zn dendrite, Zn||I_2_ aqueous battery

## Abstract

Aqueous zinc‐ion batteries (AZIBs) are attractive for grid‐scale energy storage because of their safety, low cost, and environmental benignity. However, their practical use is limited by interfacial instability at the Zn anode, which induces nonuniform Zn^2+^ flux, dendritic growth, and parasitic H_2_ evolution. Herein, we present a laser‐induced surface patterning stategy that guides Zn epitaxial growth through a resistance‐gradient‐driven mechanism. Laser ablation forms periodic concave micro‐holes, selectively removes the resistive carbonate‐based surface layer from the exposed regions, and reconstructs the Zn texture from the (101) plane to the (002) basal plane. This surface‐resistance contrast directs Zn^2+^ flux into the patterned holes, which serve as uniform nucleation sites and three‐dimensional deposition reservoirs, enabling bottom‐up Zn filling and compact homogeneous surface growth. Consequently, the patterned Zn||Zn symmetric cells achieve an exceptional lifespan of 972 cycles under high‐rate conditions (10 mA cm^−2^, 2 mAh cm^−2^). Furthermore, a 6.5 Ah Zn||I_2_ pouch cell operating at 2C (10 × 18 cm^2^, areal capacity of 3.0 mAh cm^−2^) and employing the laser‐patterned Zn anode exhibits stable cycling for over 440 cycles, a Coulombic efficiency of 99.9%, and a capacity retention of 90.7%.

## Graphical abstract

A scalable laser‐assisted surface patterning method regulates zinc ion flux and promotes lateral epitaxial growth for stable aqueous batteries. Periodic micro‐holes effectively minimize interfacial resistance and reconstruct the zinc texture to favor the compact (002) basal plane. This structured anode suppresses both dendrite propagation and parasitic reactions, enabling large‐scale pouch cells over 6 Ah to deliver superior long‐term cycling performance.

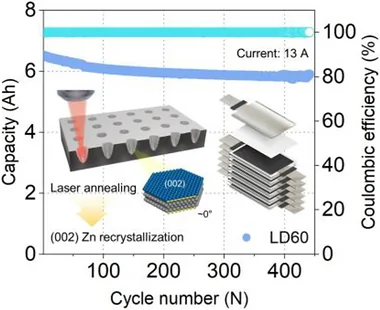

## 1 Introduction

Aqueous zinc‐ion batteries (AZIBs) have emerged as attractive candidates for grid‐scale energy storage owing to their intrinsic safety, low cost, and environmental benignity [1, 2, 3, 4]. Benefiting from the high theoretical specific capacity of Zn metal (820 mAh g^−1^) and its suitable redox potential (−0.76 V vs. SHE), AZIBs can deliver practical energy densities in the range of 60–120 Wh kg^−1^ even within the narrow electrochemical stability window of aqueous electrolytes [5, 6, 7]. Despite these advantages, the commercialization of AZIBs remains hindered by the poor reversibility of Zn metal anodes, which suffer from nonuniform Zn^2+^ flux, dendritic growth, and parasitic H_2_ evolution arising from interfacial instability [8, 9, 10]. Although Zn is generally deposited with a rounded morphology, severe dendritic growth occurs at localized current hotspots under practical operating conditions, particularly due to the high ionic conductivity of Zn^2+^ (≈50 mS cm^−1^) in aqueous electrolytes [11, 12, 13]. Moreover, the high mechanical modulus of Zn (≈100 GPa) renders Zn dendrites mechanically rigid and capable of piercing separators [14, 15]. Concurrently, the relatively low redox potential of Zn thermodynamically favors H_2_ evolution at the Zn/electrolyte interface [16]. Furthermore, the accumulation of H_2_ bubbles on the Zn surface locally blocks electroactive areas, increases the interfacial overpotential for the Zn^2+^/Zn redox reaction, and further redistributes the Zn^2+^ flux toward gas‐free regions, thereby amplifying localized dendritic growth and accelerating electrode degradation [17, 18, 19]. To mitigate these issues, extensive efforts have been devoted to stabilizing the Zn/electrolyte interface and regulating Zn electroplating/stripping behavior [20, 21].

To enhance the stability of the Zn metal/electrolyte interface, previous studies have engineered Zn surfaces to preferentially expose the (002) basal plane, which promotes a denser and more compact Zn deposition morphology than the (100) and (101) planes [22, 23, 24]. The Zn (002) plane exhibits a higher atomic packing density and lower surface energy than the (100) and (101) planes, leading to stronger electron binding and enhanced chemical stability [25, 26]. This characteristic suppresses the electron transfer required for the hydrogen evolution reaction (HER), thereby slowing HER kinetics [27]. In addition, the (002)‐textured Zn surface facilitates epitaxial Zn deposition, directing subsequent growth to maintain the same crystallographic orientation [28, 29]. Therefore, engineering Zn metal surfaces to preferentially expose the (002) texture is a key strategy for improving the electrochemical performance of AZIBs [30, 31, 32].

Laser ablation has recently emerged as a promising strategy to engineer three‐dimensional (3D) architectures on Zn metal surfaces, thereby increasing surface area and promoting more uniform Zn deposition [33, 34, 35, 36, 37]. Periodically patterned Zn electrodes with laser‐carved structures have been reported to exhibit nuclei‐rich electroplating behavior, which suppresses the formation of large protrusions and improves deposition uniformity [38]. These studies suggest that increasing the density of Zn nucleation sites through structural design is a rational pathway to mitigate dendrite formation and parasitic reactions [39, 40, 41]. However, despite recent progress, a fundamental understanding of how laser‐induced 3D architectures govern Zn crystallography and reversibility remains elusive. Specifically, the synergistic effects of surface chemistry, local resistance variations, and crystallographic reconstruction in directing Zn deposition have not yet been systematically elucidated. This lack of fundamental knowledge hinders the rational design of architectures capable of active Zn^2+^ flux regulation.

Herein, we report a rapid and scalable laser‐guided surface patterning strategy that enables controlled Zn epitaxial growth via a resistance‐gradient‐driven deposition mechanism. Laser ablation produced a regularly distributed hole structure on the Zn metal surface, featuring holes 60 µm in diameter spaced at 110 µm intervals, with tunable depth depending on the ablation parameters. The exposed fresh Zn metal at the sidewalls of the laser‐patterned holes, where initial Zn deposition occurs, serves as a preferential site for uniform Zn deposition, in contrast to the unexposed top surface. COMSOL simulations further elucidate the effects of hole geometry and local reaction kinetics on Zn deposition uniformity, identifying the optimal structural condition for compact filling without pore clogging. Owing to the synergistic effects of guided Zn^2+^ flux, 3D deposition reservoirs, and (002)‐oriented epitaxial growth, the optimized patterned Zn anode enables Zn||Zn symmetric cells to operate stably for up to 972 cycles at 10 mA cm^−2^ and 2 mAh cm^−2^. Moreover, a 6.5 Ah Zn||I_2_ pouch cell (10 × 18 cm^2^, areal capacity of 3.0 mAh cm^−2^) exhibits stable cycling for over 440 cycles while maintaining a Coulombic efficiency of 99.9% and a capacity retention of 90.7%. This work provides a practical and scalable platform for directing epitaxial Zn growth through rational 3D surface design, offering an effective route toward high‐performance aqueous Zn batteries.

## 2 Results and Discussion

Uniform Zn^2+^ plating over the entire Zn metal anode requires intrinsic surface engineering, because aqueous electrolytes inherently induce localized current concentration, which leads to severe deposition heterogeneity [42, 43]. As shown in Figure 1a, laser ablation is employed to construct a periodic hole‐patterned architecture on the Zn metal surface that effectively regulates Zn^2+^ flux and enables homogeneous Zn deposition. During laser irradiation, both the concave bottoms and the newly exposed Zn sidewalls undergo localized etching, which removes pre‐existing surface preformed layers and thereby reduces interfacial resistance relative to the pristine top surface. As shown in Figure 1b, concave patterns are precisely fabricated on Zn metal substrates using laser ablation, producing holes with a diameter of 60 µm and a center‐to‐center spacing of 170 µm. The holes were periodically arranged in both the X and Y directions to form a uniform 2D array across the Zn surface. The hole depth was systematically controlled by varying the number of laser ablation passes, where 10, 30, 50, and 70 repetitions yielded samples with average hole depths of approximately 20, 60, 100, and 140 µm, respectively, denoted as LD20, LD60, LD100, and LD140. During fabrication, the total thickness of the Zn substrate was fixed at 200 µm, which ensured consistent mechanical stability and provided sufficient margin for the concave pattern depth, thereby preventing microcrack formation during laser processing (Figure S1). The hole‐patterned Zn surface significantly modified electrolyte wettability, primarily through a capillary‐driven wetting effect. The measured water contact angles for bare Zn, laser‐hatched Zn (HC, where laser irradiation was only applied to the surface without forming a concave structure), LD20, LD60, LD100, and LD140 were 89°, 84°, 72°, 33°, 28°, and 131°, respectively (Figure 1c and Figure S2). Notably, the bare Zn and HC surfaces exhibited similar apparent water contact angles, indicating that laser irradiation without concave geometries had a negligible impact on intrinsic surface wettability. In contrast, the introduction of hole patterns improved electrolyte wetting, which was attributed to the strong capillary pressure generated within the concave microstructures that promoted spontaneous electrolyte infiltration [44]. The laser‐induced hole patterns acted as microcapillaries that drove electrolyte infiltration via capillary pressures of 1.44, 3.91, 4.12, and −3.06 kPa for LD20, LD60, LD100, and LD140, respectively, as detailed in the supplementary information (Figure S3) [45]. Notably, LD140 exhibited the largest apparent water contact angle, which was attributed to the formation of trapped air pockets within the deep cavities, consistent with the Cassie–Baxter wetting model [46, 47, 48]. In contrast, for shallower hole depths, electrolyte infiltration into the concave structures increased the effective solid–liquid contact area and enabled more uniform electrolyte spreading over the patterned surface. Consequently, capillary forces generated within the holes facilitated rapid electrolyte uptake and reduced the apparent water contact angle, thereby enhancing interfacial wettability and promoting a more homogeneous Zn^2+^ ion distribution during electrochemical cycling.

**FIGURE 1:**
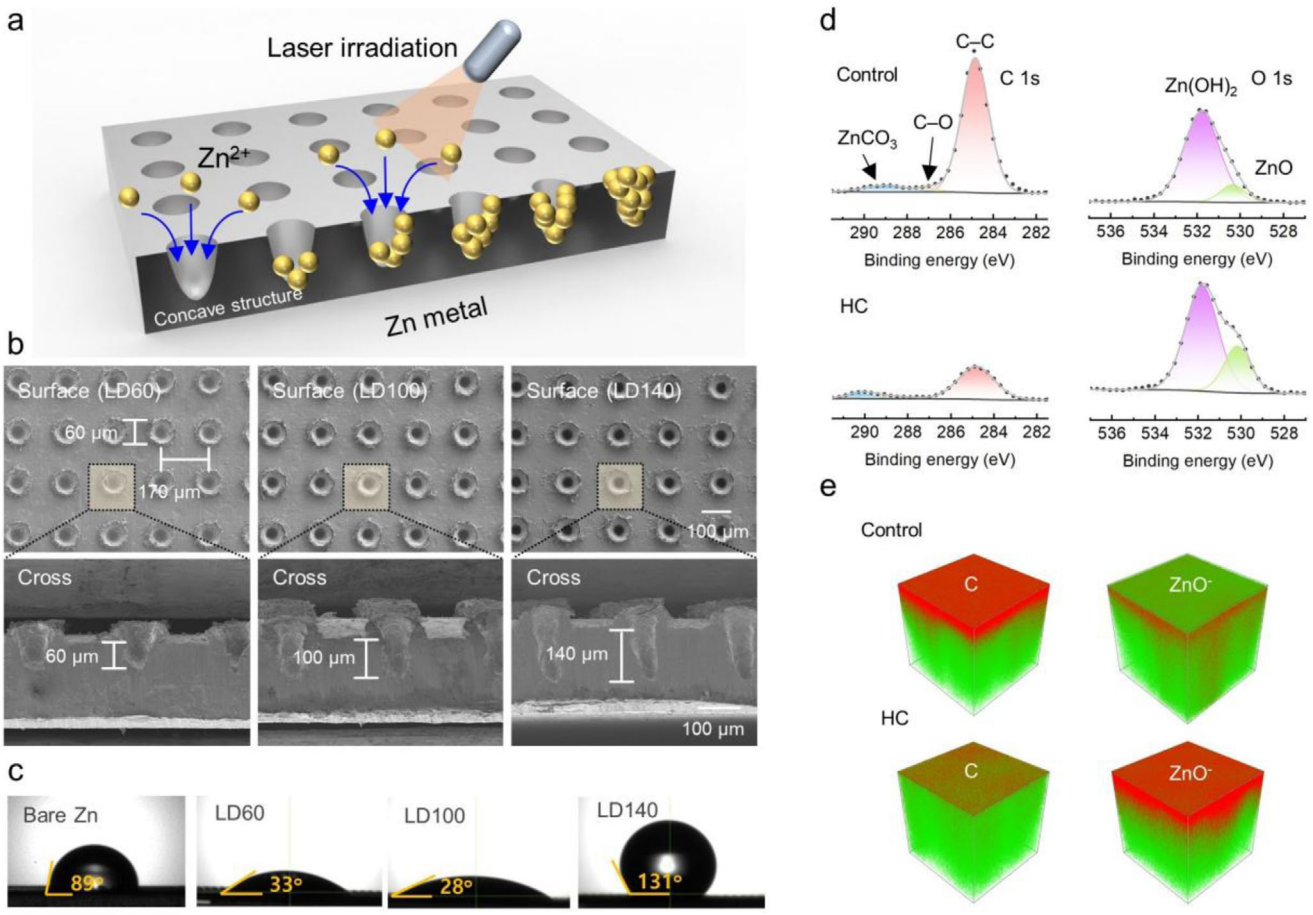
(a) Schematic of Zn^2+^ ion deposition behavior on a laser‐patterned Zn metal anode. (b) SEM images of the laser‐exposed Zn metal anode surface and corresponding cross‐sectional views showing pattern depths of 60, 100, and 140 µm. (c) Water contact angle measurements of a 2 M ZnSO_4_ aqueous electrolyte on bare Zn (Control) and laser‐patterned Zn anodes with depths of 60 (LD60), 100 (LD100), and 140 µm (LD140). (d) XPS analysis of the Control and laser‐hatched Zn (HC) electrodes, including C 1s and O 1s spectra. (e) TOF‐SIMS depth profiling of the Control and HC surfaces.

As shown in Figure 1d, the XPS C 1s spectra of bare Zn exhibit an intense C–C signal attributed to carbonate species formed during production, whereas the HC electrode shows a significantly attenuated C–C peak [49, 50, 51]. Correspondingly, the O 1s spectra reveal a lower ZnO content on the bare Zn surface, while the HC electrode displayed a markedly higher ZnO intensity [52, 53]. The laser‐exposed HC Zn exhibited lower interfacial resistance and stripping/plating polarization than untreated Zn (Figures S4 and S5). At 5 mA cm^−2^, the initial stripping polarization decreased from 105 to 57 mV, while the SEI and charge‐transfer resistances decreased from 85.74 to 19.68 Ω and from 246 to 71.89 Ω, respectively. This resistance contrast redistributed current toward the concave regions, promoting preferential Zn^2+^ flux and Zn deposition within the laser‐patterned recesses. Consistent with these results, Tafel analysis showed that the exchange current, obtained by extrapolating the fitted Tafel line to zero overpotential, increased from 2.05 × 10^−3 ^mA for the Control to 9.51 × 10^−3^ mA for HC, corresponding to an approximately 4.6‐fold enhancement (Figure S6). The initial Zn seeds formed within these holes subsequently templated continued deposition, leading to more uniform plating behavior. Furthermore, time of flight secondary ion mass spectrometry (TOF‐SIMS) depth profiling provided insights into the compositional distribution of the Zn metal from the top surface to the inner region (Figure 1e). Consistent with the XPS results, the bare Zn surface exhibited a high concentration of carbonate‐based compounds on the surface alongside low ZnO content. In contrast, the HC electrode displayed a significantly reduced presence of resistive carbonates and a markedly higher ZnO concentration at the surface. Furthermore, laser irradiation locally raised the Zn surface temperature to approximately 800–1000 °C, inducing partial melting. During subsequent cooling, the molten layer recrystallized to minimize its total surface energy. Because the close‐packed Zn(002) basal plane has a lower specific surface energy than Zn(101), (002)‐oriented grains are thermodynamically favored during resolidification [27, 54]. Consequently, the I_(002)_/I_(101)_ intensity ratio increased from 0.73 for bare Zn, which predominantly exhibited the (101) orientation, to 0.94 for HC (Figure S7). The influence of the initial lattice modification on subsequent Zn deposition behavior will be discussed later.

We systematically investigated the Zn deposition behavior on the LD60 electrode at a constant current density of 5 mA cm^−2^, while varying the areal capacity from 1 to 10 mAh cm^−2^. As shown in Figure 2a, the bare Zn electrode exhibits highly irregular deposition at a capacity of 5 mAh cm^−2^. Both top‐view and cross‐sectional analyses revealed non‐uniform thickness and localized aggregates, reflecting the uneven Zn^2+^ flux and uncontrolled nucleation that occurred during plating on unmodified surfaces. In contrast, Zn was preferentially deposited within the hole cavity rather than on the planar surface at areal capacities of 1 and 3 mAh cm^−2^ (Figure 2b,c). This selective deposition originated from the reduced interfacial resistance of the laser‐exposed sidewalls, where the resistive native carbonate layer was effectively removed during ablation [36]. Consequently, this resistance gradient directed the Zn^2+^ flux toward the recessed regions, facilitating a bottom‐up filling mechanism that suppressed uncontrolled surface growth and ensured a more uniform morphology. As the deposition capacity was increased from 5 to 10 mAh cm^−2^ (Figure 2d,e), the hole cavity reached near full occupancy. Once the cavities were filled, subsequent Zn deposition preferentially occurred on the planar surfaces between the laser‐patterned regions. Furthermore, when comparing the Zn deposition morphology with the naked eye, the bare Zn electrode exhibited markedly uneven and irregular deposits. In contrast, the laser‐patterned electrodes, regardless of the deposited capacity, showed uniform and coherent Zn coverage. Notably, the deposited surface color gradually darkened as the capacity increased from 1 to 10 mAh cm^−2^, indicating progressive and continuous Zn accumulation guided by the patterned structure.

**FIGURE 2:**
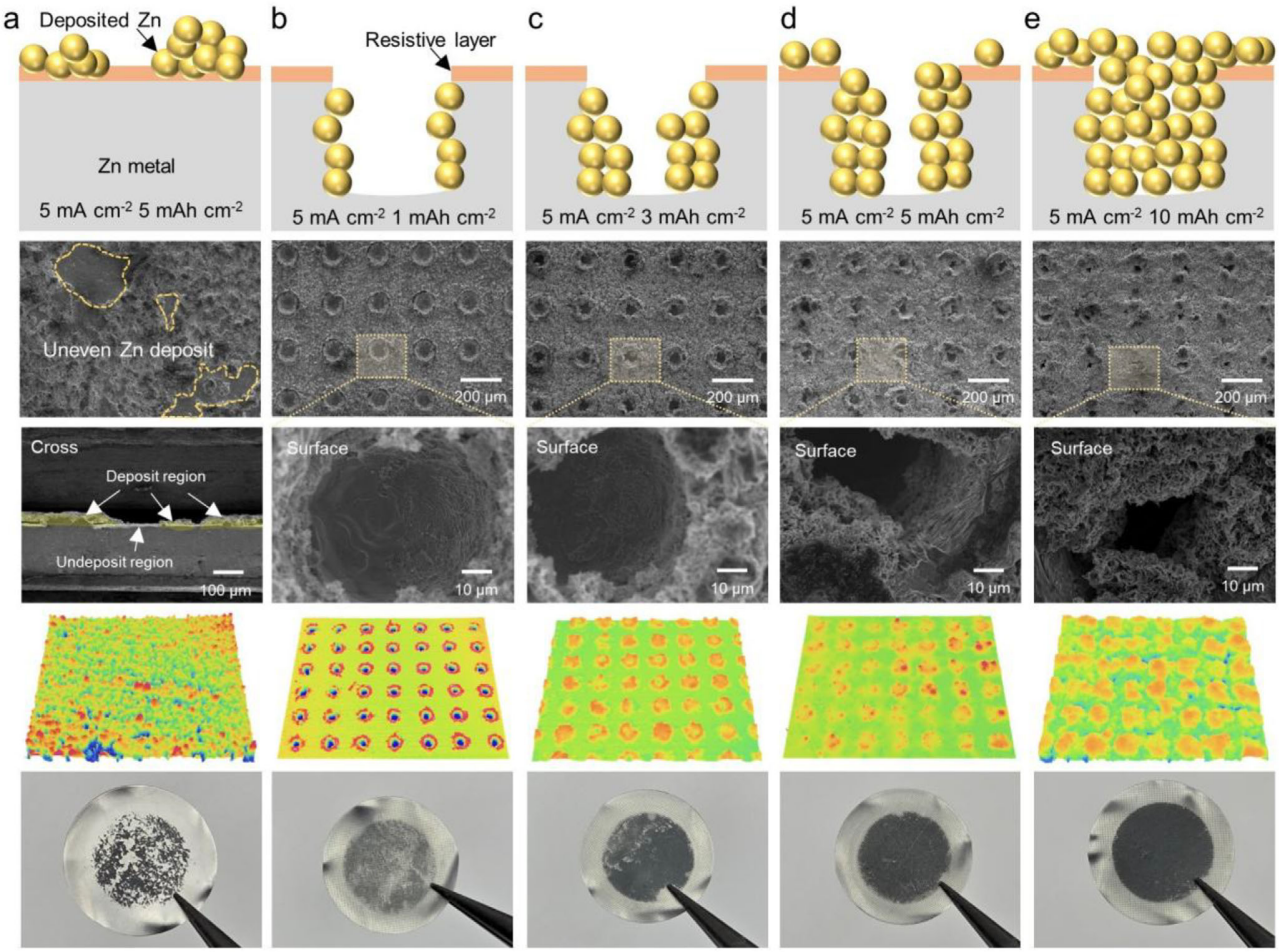
Comparison of Zn deposition behavior under a current density of 5 mA cm^−2^ using schematics, SEM images, confocal 3D images, and digital photographs. Zn plating was conducted on (a) bare Zn with a capacity of 5 mAh cm^−2^ and on LD60 at areal capacities of (b) 1 mAh cm^−2^, (c) 3 mAh cm^−2^, (d) 5 mAh cm^−2^, and (e) 10 mAh cm^−2^ respectively.

Figure S8 shows the Zn stripping behavior at various areal capacities ranging from 1 to 10 mAh cm^−2^ under a current density of 5 mA cm^−2^. The bare Zn electrode exhibited incomplete stripping with noticeable unreacted regions and a rough, irregular surface morphology after stripping at 5 mAh cm^−2^. In contrast, for the LD60 electrode, stripping initiated predominantly from the previously deposited Zn within the hole structures. As the stripping capacity increased to 10 mAh cm^−2^, the hole structures became distinctly visible, indicating that most of the pre‐deposited Zn within the cavity was removed first, followed by stripping of the residual Zn from the top surface outside the patterned regions. These results demonstrate that the 3D hole patterns provide a guiding framework for reversible and localized plating and stripping of the Zn metal anode.

To validate the efficacy of laser patterning on Zn metal surfaces, elucidating the microscopic behavior underlying Zn deposition is essential. Therefore, we used phase‐field modeling to investigate the electrochemical deposition behavior (Figure 3). The detailed simulation setup, including the model geometry and boundary conditions, is provided in Figure S9. The chemical and morphological heterogeneity of the Zn surface was modeled via distributed activated/deactivated sites. In the Control model, the chemically and microscopically heterogeneous Zn surface induced localized Zn nucleation, resulting in a bumpy and dendritic morphology (Figure 3a and Video S1). Such uncontrolled dendritic growth leads to substantial volume expansion and may induce internal short circuits, thereby deteriorating the structural and electrochemical stability of the cell [55, 56]. As was shown in Figure 1, removal of the pre‐existing surface layer via laser ablation reduced the interfacial resistance, meaning that the exchange current density at the hole surface (*i*_0,hole_) was higher than that of the bare Zn surface (*i*_0_) [14, 36]. To examine the effect of the resistance gradient induced by laser ablation, simulations were conducted by systematically varying *i*_0,hole_ with a fixed hole depth of 60 µm. The *i*_0,hole_ = 3 *i*_0_ condition was included as an intermediate comparison, whereas *i*_0,hole_ = 5 *i*_0_ was based on an experimental estimate from the impedance measurements (see Methods for details). When *i*_0,hole_ was equal to *i*_0_, Zn nucleation simultaneously occurred on the surface and inside the holes, and the resulting localized Zn growth led to pronounced dendritic formation after deposition at 10 mAh cm^−2^ (Figure 3b and Video S2). Increasing *i*_0,hole_ to three times that of *i*_0_ promoted Zn deposition within the holes, which alleviated dendritic growth originating from localized nucleation at the surface and led to a more uniform morphology (Figure S10). When *i*_0,hole_ was further increased to five times that of *i*_0_, Zn deposition on the top surface was markedly reduced, producing an exceptionally smooth morphology (Figure 3c and Video S3). This effect originated from enhanced reaction kinetics at the hole surfaces, which locally accelerated Zn deposition and directed Zn growth into the concave regions, thereby enabling bottom‐up filling within the holes. In contrast, when *i*_0,hole_ was lower than *i*_0_, Zn deposition inside the holes became sluggish, whereas deposition on the top surface became dominant, resulting in pronounced dendritic growth and delayed hole filling (Figure S11). This bottom‐up filling behavior was further supported by quantitative analysis of the hole porosity (Figure 3d). When *i*_0,hole_ was higher than *i*_0_, Zn deposition was directed into the holes, leading to a rapid decrease in hole porosity as deposition proceeded. In contrast, when *i*_0,hole_ was comparable or lower than *i*_0_, Zn deposition on the top surface and within the holes was comparable, resulting in a much slower reduction in hole porosity.

**FIGURE 3:**
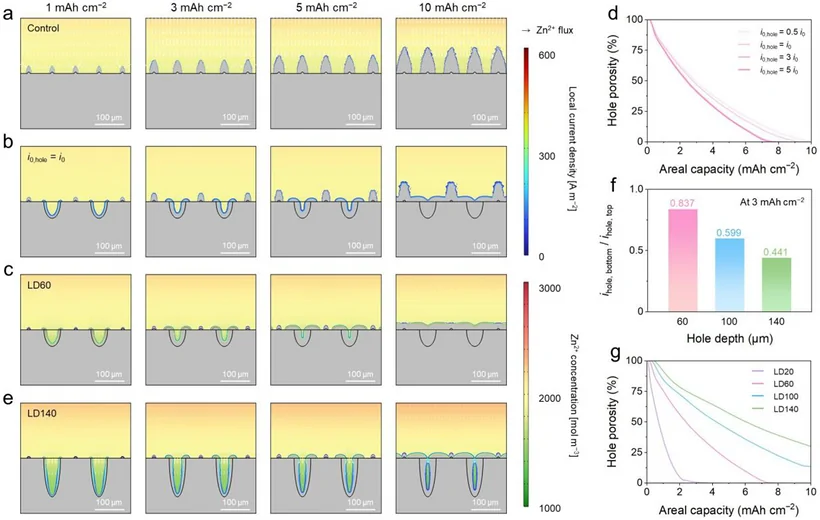
Phase‐field simulation results of electrodeposition at various areal capacities on (a) bare Zn (Control), (b) patterned Zn with *i*_0,hole_ = *i*_0_, and (c) LD60. (d) Evolution of hole porosity with areal capacity under varying *i*_0_ values. (e) Phase‐field simulation results of electrodeposition at various areal capacities on LD140. (f) Ratio of minimum to maximum local current density as a function of hole depth at 3 mAh cm^−2^. (g) Evolution of hole porosity with areal capacity under varying hole depths.

Having established that preferential Zn deposition occurred when the hole surfaces exhibited lower interfacial resistance than the bare Zn surface, we next investigated the influence of hole depth with *i*_0,hole_ fixed at five times that of *i*_0_. For LD20, Zn deposition preferentially occurred within the holes without leaving internal cavities; however, the shallow hole provided only a relatively small storage capacity for Zn deposition, thereby limiting the mitigation of volume expansion (Figure S12). Increasing the depth to 60 µm (LD60) enabled compact and uniform filling within the holes, producing a smooth surface morphology without residual porosity (Figure 3c). However, when the depth was further increased to 100 µm (LD100), Zn deposition began to accumulate near the hole entrance, leaving partially unfilled cavities inside the holes (Figure S13). As shown in Figure 3e and Video S4, LD140 exhibits a similar blocking behavior with more pronounced internal cavities remaining within the holes due to stronger localization of Zn deposition near the hole entrance. Figure 3f shows the current distribution along the hole depth at a plating capacity of 3 mAh cm^−2^, revealing that the normalized current at the hole bottom decreases markedly with increasing hole depth. This indicates that deeper holes induce strong current localization near the hole entrance, which suppresses Zn deposition at the bottom of the holes and eventually leads to pore clogging. Consistently, the evolution of hole porosity shows that LD20 and LD60 approach complete filling during deposition, whereas LD100 and LD140 retain residual porosity even at high areal capacities due to pore clogging (Figure 3g). Consequently, the phase‐field analysis identified LD60 with high *i*_0,hole_ as an optimal hole depth condition that was sufficiently deep to mitigate volume expansion while remaining shallow enough to enable compact Zn filling without residual cavities.

We evaluated the interfacial stability of Zn symmetric cells assembled with bare Zn (Control), HC, and laser‐patterned Zn electrodes (LD20, LD60, LD100, and LD140). As shown in Figure 4a, the Control and HC electrodes fail rapidly after 52 h (130 cycles) and 198 h (495 cycles), respectively, reflecting severe interfacial instability. In contrast, the patterned electrodes delivered markedly extended lifetimes of 192 h (480 cycles), 1114 h (2785 cycles), 828 h (2070 cycles), and 606 h (1515 cycles) for LD20, LD60, LD100, and LD140, respectively, under 5 mA cm^−2^ with an areal capacity of 1 mAh cm^−2^. When the areal capacity was increased to 3 mAh cm^−2^ at 5 mA cm^−2^ (Figure 4b), the Control, HC, LD20, LD60, LD100, LD140 electrodes showed lifetimes of 68 h (56 cycles), 93 h (77 cycles), 148 h (123 cycles), 310 h (258 cycles), 196 h (163 cycles), and 128 h (106 cycles), respectively. While the Control (17 h = 42 cycles) and HC (49 h = 48 cycles) electrodes showed poor stability under harsh conditions (10 mA cm^−2^, 2 mAh cm^−2^), the laser‐patterned electrodes (LD20, LD60, LD100, and LD140) still exhibited significantly enhanced stability, delivering lifetimes of 155 h (388 cycles), 389 h (972 cycles), 284 h (710 cycles), and 182 h (455 cycles), respectively (Figure 4c). These results indicate that laser‐induced surface modification alone (HC) was insufficient for substantial stability enhancement. Instead, the electrode with an optimized patterning depth of 60 µm (LD60) delivered the best electrochemical performance. Notably, the LD60 electrode achieved state‐of‐the‐art interfacial stability, which was comparable to previously reported Zn||Zn symmetric cell in terms of current density, areal capacity, and cycle life (Figure S14 and Table S2).

**FIGURE 4:**
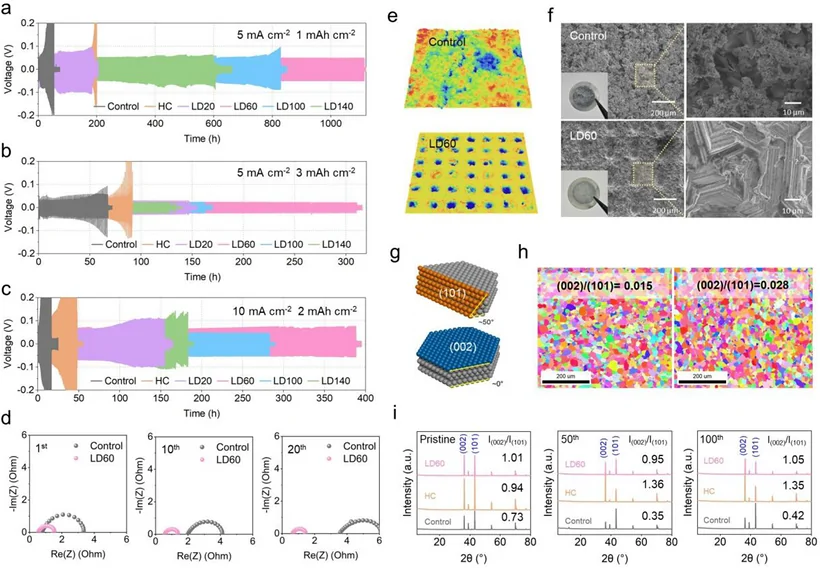
Electrochemical performance of Zn||Zn symmetric cells using Control, HC, LD20, LD60, LD100, and LD140 electrodes. Galvanostatic cycling profiles under (a) 5 mA cm^−2^ and 1 mAh cm^−2^, (b) 5 mA cm^−2^ and 3 mAh cm^−2^, and (c) 10 mA cm^−2^ and 2 mAh cm^−2^. (d) EIS spectra of Zn||Zn symmetric cells after 1, 10, and 20 cycles for the Control and LD60 electrodes. Comparison of the Zn metal surface in Zn||Zn symmetric cells at the plated state after 50 cycles (10 mA cm^−2^, 2 mAh cm^−2^): (e) 3D confocal images and (f) SEM images of the Control and LD60 electrodes. (g) Representative images of Zn lattice orientations corresponding to the (101) and (002) planes. (h) EBSD maps of the Control and LD60 electrodes after 50 cycles in Zn||Zn symmetric cells. (i) XRD patterns of the Control, HC, and LD60 electrodes in the pristine state and after 50 and 100 cycles, compared based on the I_(002)_/I_(101)_ peak intensity ratio.

The electrochemical impedance spectroscopy (EIS) curves shown in Figure 4d reveal a progressive increase in interfacial impedance for both the Control and LD60 electrodes after repeated cycling (1, 10, and 20 cycles). Notably, the Control electrode exhibited a substantially larger increase in both ohmic resistance (*R*_ohm_) and charge transfer resistance (*R*_ct_) than the LD60 electrode, indicating that the laser‐induced surface architecture effectively suppressed interfacial degradation and preserved stable Zn^2+^ ion transport during cycling. The pronounced resistance growth observed for the Control electrode was primarily attributed to the continuous accumulation of porous, resistive byproduct layers on the Zn surface that electrically passivated the underlying metal and impeded reversible Zn plating/stripping. In contrast, the patterned Zn electrode maintained stable interfacial resistance even after 50, 100, and 300 cycles, demonstrating its superior interfacial stability (Figure S15).

Figure 4e shows the Zn deposition morphology of the Control and LD60 electrodes, analyzed by 3D confocal microscopy after 50 cycles in Zn||Zn symmetric cells. The Control electrode exhibited extensive accumulation of mossy Zn deposits with a highly porous and uneven surface, indicative of uncontrolled Zn growth. In contrast, the LD60 electrode displayed a much more compact and dense morphology across both the concave hole regions and the laser‐unexposed top surface. The concave pattern remained discernible after 300 cycles, indicating that repeated Zn plating and stripping did not completely fill or erase the features (Figure S16). The other laser‐patterned electrodes with different pattern depths also exhibited highly uniform Zn deposition morphologies in 3D confocal image analysis (Figure S17). Notably, in the SEM images shown in Figure 4f, the deposited Zn on the LD60 electrode exhibits a distinctive comb‐like textured morphology that is markedly different from that observed on the Control electrode. Although the patterned holes were largely filled with deposited Zn, traces of the original hole geometry remained discernible, suggesting regulated and confined Zn growth within the patterned architecture. As shown in Figure S18, the Zn deposition morphology evolves consistently across different patterning depths. Unlike the Control electrode, all patterned surfaces promoted compact Zn growth, with the laser‐treated areas guiding the resulting surface structure. The detailed 3D surface profiles and line scans shown in Figure S19 reveal that, even after 50 cycles, the LD60 electrode preserves its periodic concave structure with Zn deposition confined within the patterned geometry, whereas the Control electrode exhibits a highly irregular surface with large height fluctuations caused by inhomogeneous Zn protrusions. As depicted in Figure 4g, the Control electrode shows vertically oriented dendritic Zn deposits, which are associated with growth along the (101) plane, whereas the LD60 electrode exhibits laterally compact deposits that are indicative of preferential growth along the (002) basal plane [54].

To quantify the crystallographic orientation of the deposited Zn, surface electron backscatter diffraction (EBSD) analysis was performed (Figure 4h). The Control electrode exhibited a I_(002)_/I_(101)_ ratio of 0.015, whereas the LD60 electrode yielded a nearly twofold increase in this ratio to 0.028. This enrichment of the (002) plane demonstrated that the laser‐patterned architecture effectively promoted the growth of the (002) lattice, which is well‐documented to enhance interfacial stability in Zn metal anodes. Figure 4i shows the X‐ray diffraction (XRD) patterns of the Control, HC, and LD60 electrodes before and after 50 and 100 cycles, which were analyzed to investigate the evolution of Zn crystallinity and to determine whether the initially laser‐exposed Zn regions, dominated by the (002) lattice orientation, influenced subsequent Zn deposition. The Control electrode exhibited an initial I_(002)_/I_(101)_ intensity ratio of 0.73, which decreased to 0.35 and 0.42 after 50 and 100 cycles, respectively, indicating the dominant development of the (101) lattice during repeated cycling. In contrast, the HC and LD60 electrodes showed higher initial I_(002)_/I_(101)_ ratios of 0.94 and 1.01, respectively, which changed to 1.36 and 0.95 after 50 cycles and to 1.35 and 1.05 after 100 cycles, demonstrating that the patterned Zn electrodes maintained or enhanced the preferential (002)‐oriented crystallographic texture. This behavior is attributed to homoepitaxial Zn growth, in which nuclei aligned with the underlying Zn(002) grains form coherent, low‐mismatch interfaces. These interfaces reduce the interfacial energy and nucleation barrier relative to randomly oriented nuclei, thereby promoting reversible (002) oriented deposition [28].

The HER behaviors of the Control and LD60 electrodes were investigated at a current density of 10 mA cm^−2^ to evaluate the role of guided Zn deposition in suppressing parasitic gas evolution. For the Control electrode, pronounced gas bubble formation was observed from 10 min, followed by rapid bubble growth and accumulation on the Zn surface with prolonged polarization (Figure 5a). These bubbles locally blocked ionic transport pathways and induced highly heterogeneous Zn^2+^ flux, which further accelerated uneven Zn deposition and localized dendritic growth. In contrast, the laser‐patterned Zn electrode exhibited markedly suppressed bubble nucleation throughout the entire observation period up to 60 min (Figure 5b). Furthermore, optical microscopy in a 1 × 1 cm^2^ beaker cell showed that, after 50 cycles at 10 mA cm^−2^ and 2 mAh cm^−2^, the Control electrode accumulated large H_2_ bubbles and developed bare non‐plated regions, whereas the patterned electrode showed negligible H_2_ evolution, confirming its superior interfacial stability (Figure S20). Linear sweep voltammetry (LSV) curves were used to evaluate the HER onset potentials for the Control and LD60 electrodes (Figure 5c), recorded at −1.40 and −1.52 V (vs. Ag/AgCl), respectively. Notably, at cathodic current densities beyond −5 mA cm^−2^, the LD60 electrode reached a more negative potential (−1.80 V) than the Control electrode (−1.67 V). This behavior suggested that the (002) plane possessed a higher atomic packing density and lower surface energy than alternative planes; this configuration resulted in a stronger electron binding affinity, which inhibited the electron‐donating reactions essential for the HER and effectively slowed its reaction kinetics [57]. To quantitatively assess the intrinsic HER kinetics, Tafel polarization measurements were conducted (Figure 5d). The measured corrosion current densities (*I*_corr_) were 0.0287 and 0.0118 mA cm^−2^ for the Control and LD60 electrodes, respectively, with corresponding corrosion potentials (*E*_corr_) of −985 and −979 mV. These results indicated that the laser‐exposed patterned layer, which contained a higher fraction of oriented Zn(002) planes, effectively mitigated corrosion by regulating interfacial reactions.

**FIGURE 5:**
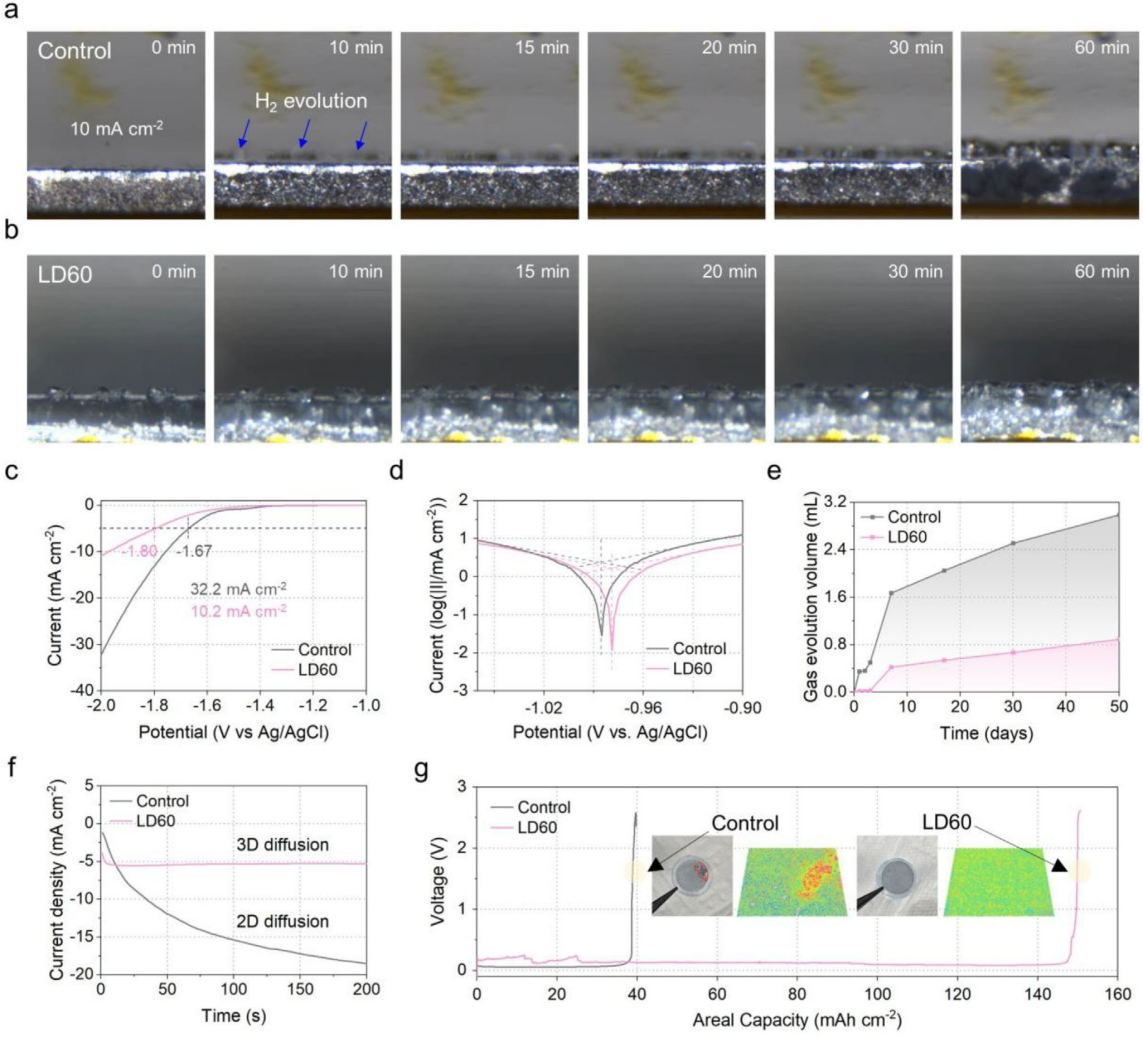
Analysis of HER behavior on Zn metal at a current density of 10 mA cm^−2^ and various plating times for the (a) Control and (b) LD60 electrodes. (c) LSV curves of the Control and LD60 electrodes measured in 1 M aqueous Na_2_SO_4_ at a scan rate of 1 mV s^−1^. (d) Tafel plots of the Control and LD60 Zn anodes. (e) Comparison of the H_2_ evolution volume during storage for the Control and LD60 electrodes. (f) Chronoamperometric current response of a Zn||Zn symmetric cell with Control and LD60 electrodes at an applied potential of −150 mV for 200 s. (g) Short‐circuit stability test of the Control and LD60 electrodes conducted at a current density of 20 mA cm^−2^.

The cumulative H_2_ evolution during aging was quantified by measuring the total H_2_ gas volume as a function of storage time (Figure 5e). The Control electrode packaged in a 3 × 3 cm^2^ pouch cell exhibited continuous and accelerated H_2_ evolution, reaching 3.0 mL over 50 days, which indicated persistent parasitic reactions even under resting conditions. In contrast, the LD60 electrode exhibited a substantially lower gas evolution rate, with a total accumulated H_2_ volume of only 0.85 mL. This suppressed gas evolution was also macroscopically evident in the significantly reduced swelling of the LD60 pouch cell compared to the Control cell after long‐term storage (Figure S21). As shown in Figure S22, the Control Zn||Zn symmetric cell exhibits a pronounced increase in semicircle diameter during 0–48 h of aging, whereas the LD60 cell shows only a slight increase, indicating that the laser‐patterned structure effectively suppresses H_2_ evolution and surface corrosion during storage. To investigate Zn nucleation and growth kinetics, chronoamperometric measurements were performed at a constant overpotential of −150 mV for 200 s (Figure 5f). The Control electrode showed a continuously increasing negative current, indicating the sustained 2D diffusion of Zn^2+^ ions throughout the entire deposition process, leading to uncontrolled Zn dendrite growth. In contrast, the LD60 electrode demonstrated an initial transient 2D diffusion phase, followed by a prolonged 3D compact diffusion process. These results indicated that the 3D hole‐patterned architecture effectively regulated Zn^2+^ transport and localized ion reduction within the patterned regions, thereby suppressing lateral diffusion and guiding subsequent deposition toward dense, confined Zn growth (Figure S23). Figure 5g shows a comparison of the short‐circuit stability of the Control and LD60 electrodes under continuous Zn plating at a current density of 20 mA cm^−2^. The Control cell exhibited a sudden voltage drop and short‐circuit at approximately 40 h, indicative of internal failure caused by uncontrolled dendritic growth. In contrast, the LD60 cell maintained stable polarization without short‐circuiting for over 150 h, demonstrating the effectiveness of guided Zn deposition in suppressing dendrite propagation.

As shown in Figure 6a, Zn||I_2_ coin‐type cells operated at a high rate of 4C (= 14.4 mA cm^−2^) exhibited enhanced cycling stability when employing the Control and LD60 electrodes. The Control electrode showed progressive capacity decay, stemming from severe interfacial instability and unregulated dendritic growth, which ultimately led to an internal short circuit at approximately the 501st cycle. Conversely, the LD60 electrode exhibited superior stability, retaining 67% of its initial 3.58 mAh cm^−2^ capacity after 5894 cycles with an average Coulombic efficiency of 99.9%. The corresponding voltage profiles at certain cycle numbers revealed that cells employing bare Zn exhibited a gradual capacity decline and ultimately a short‐circuit event was observed during the charging process at the 501st cycle (Figure 6b). In contrast, cells employing LD60 maintained stable charge/discharge plateaus for over 4000 cycles without any short‐circuit behavior, demonstrating its superior interfacial stability and cycling durability (Figure 6c). After 500 cycles, the Control Zn electrode exhibited substantial accumulation of zinc hydroxide sulfate (ZHS, Zn_4_(OH)_6_SO_4_·xH_2_O). ZHS can form when OH^−^ generated by the hydrogen evolution reaction reacts with Zn^2+^ and SO_4_^2−^ in the electrolyte (Figures S24, S25). In contrast, the LD60 electrode showed much less ZHS deposition, and its concave features remained largely unobstructed. We further examined the stability of the Zn anodes in an I3^−^ containing electrolyte. After immersion in 2 M ZnSO_4_ containing 0.1 M I_3_^−^, the LD60 electrode retained a smoother surface than the Control electrode (Figure S26). EIS measurements of Zn||Zn symmetric cells stored without cycling in the same electrolyte also showed a smaller increase in impedance for LD60 (Figure S27). These results indicate that LD60 undergoes less surface degradation during exposure to the I_3_^−^ containing electrolyte. Moreover, polyiodide crossover and the associated Zn corrosion can contribute to self‐discharge. When cells were fully charged and then rested for 48 h, the Control cell retained 90.3% of its capacity, whereas the LD60 cell retained 96.1% (Figure S28), indicating that LD60 effectively suppresses polyiodide shuttling and the resulting parasitic reactions. The electrochemical performance of Zn||I_2_ full cells at a rate of 10C showed that LD60 delivered the most stable cycling retention among all electrode variants, including HC, LD20, LD100, and LD140 (Figure S29). Furthermore, rate capability tests conducted over a wide range of current densities from 0.2 to 10C demonstrated that the LD60 electrode delivered consistently higher reversible capacity (Figure 6d). As shown in Figure 6e,f, the LD60 electrode exhibits significantly reduced voltage polarization compared to the Control electrode across a wide range of C‐rates. At a rate of 10C, the disparity in discharge capacity became particularly pronounced. This demonstrated the effectiveness of the 3D‐patterned structure in mitigating localized current concentrations, facilitating rapid Zn^2+^ transport, and suppressing kinetic polarization during high‐rate charging and discharging.

**FIGURE 6:**
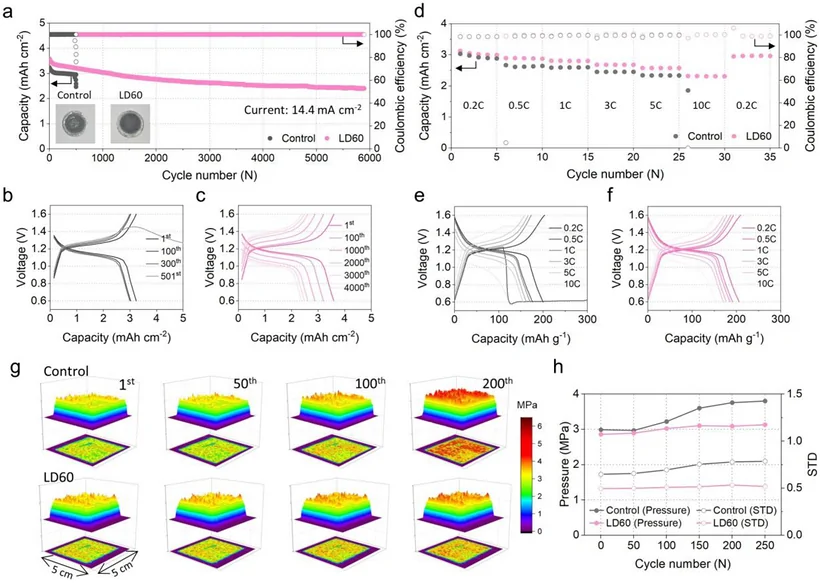
(a) Electrochemical cycling performance of Zn||I_2_ coin‐type cells operated at a rate of 4C using Control and LD60 electrodes. Voltage profiles of Zn||I_2_ cells at selected cycle numbers for the (b) Control and (c) LD60 electrodes. (d) Rate capability of Zn||I_2_ cells measured at 0.2C, 0.5C, 1C, 5C, 10C, and 20C using Control and LD60 electrodes. Voltage profiles of Zn||I_2_ cells measured at every third cycle under stepwise rate conditions (0.2C, 0.5C, 1C, 3C, 5C, and 10C) for the (e) Control and (f) LD60 electrodes. (g) Comparison of the pressure distribution in 5 × 5 cm^2^ Zn||I_2_ pouch cells using Control and LD60 electrodes after 1, 50, 100, and 200 cycles. (h) Comparison of the average pressure and pressure STD for Zn||I_2_ pouch cells using Control and LD60 electrodes during cycling.

To confirm that cell failure originated from inhomogeneous, dendritic Zn growth, we monitored the in situ pressure distribution on the Zn anode during cycling using a pressure mapping system (Figure 6g). The Control cell showed a relatively uniform pressure profile after 1 cycle; however, pronounced pressure heterogeneity developed after 50, 100, and 200 cycles, indicating localized Zn accumulation and stress concentration. In contrast, the LD60 cell maintained a much more homogeneous pressure distribution up to 200 cycles, consistent with uniform Zn plating. Figure 6h shows the internal average pressure and standard deviation (STD) of the bare Zn||I_2_ and LD60//Zn||I_2_ full cells as a function of cycle number, providing quantitative insights into the uniformity of Zn plating. For the bare Zn electrode, the initial average pressure of 3.0 MPa increased significantly to approximately 3.8 MPa by the 250th cycle. This substantial increase was accompanied by a jump in the STD value from an initial approximately 0.64 to over 0.79, indicating highly non‐uniform pressure generation across the cell. This pressure instability was primarily attributed to H_2_ evolution via the HER and the formation of uneven byproducts on the Zn surface [43, 58, 59]. These trends confirmed that the bare Zn electrode suffered from severe parasitic reactions and poor mechanical stability, leading to an increasingly heterogeneous reaction environment. In contrast, the LD60 electrode exhibited much higher mechanical stability, with the average pressure increasing only slightly from 2.8 MPa initially to 3.1 MPa at the 250th cycle. Furthermore, the STD value remained nearly constant, changing only slightly from 0.49 to 0.51. This minimal variation demonstrated that the patterned architecture maintained a uniform interfacial environment and effectively suppressed parasitic gas evolution.

Finally, we demonstrated the scalability of Zn||I_2_ pouch cells by evaluating cells of various sizes to verify their feasibility for large‐format implementation. For the large‐format Zn||I_2_ pouch cells, carbon‐based cathodes were first fabricated using a solvent‐free dry‐electrode process, followed by three‐roll milling and roll‐to‐roll processing with a width of 14 cm (Figures S30–S32). Iodine active material was subsequently introduced into the pre‐formed dry carbon electrodes through an impregnation process, enabling uniform iodine loading within the porous cathode structure. Figure 7a shows the cycling performance of the 0.5 Ah pouch cell (6 × 12 cm^2^) employing the LD60 electrode, which achieves 86.5% capacity retention over approximately 678 cycles with a Coulombic efficiency above 99.8%. In contrast, the 0.5 Ah pouch cell employing bare Zn retained 80% of its capacity for only 320 cycles, corresponding to less than half the cycle life achieved by the pouch cell employing the LD60 electrode. As shown in Figures S33 and S34, the Control electrode exhibits non‐uniform Zn deposition, dendritic penetration, and severe H_2_ gas accumulation inside the pouch cell upon failure. In contrast, the LD60 electrode displayed a highly uniform Zn morphology, although the cell ultimately failed due to electrolyte depletion.

**FIGURE 7:**
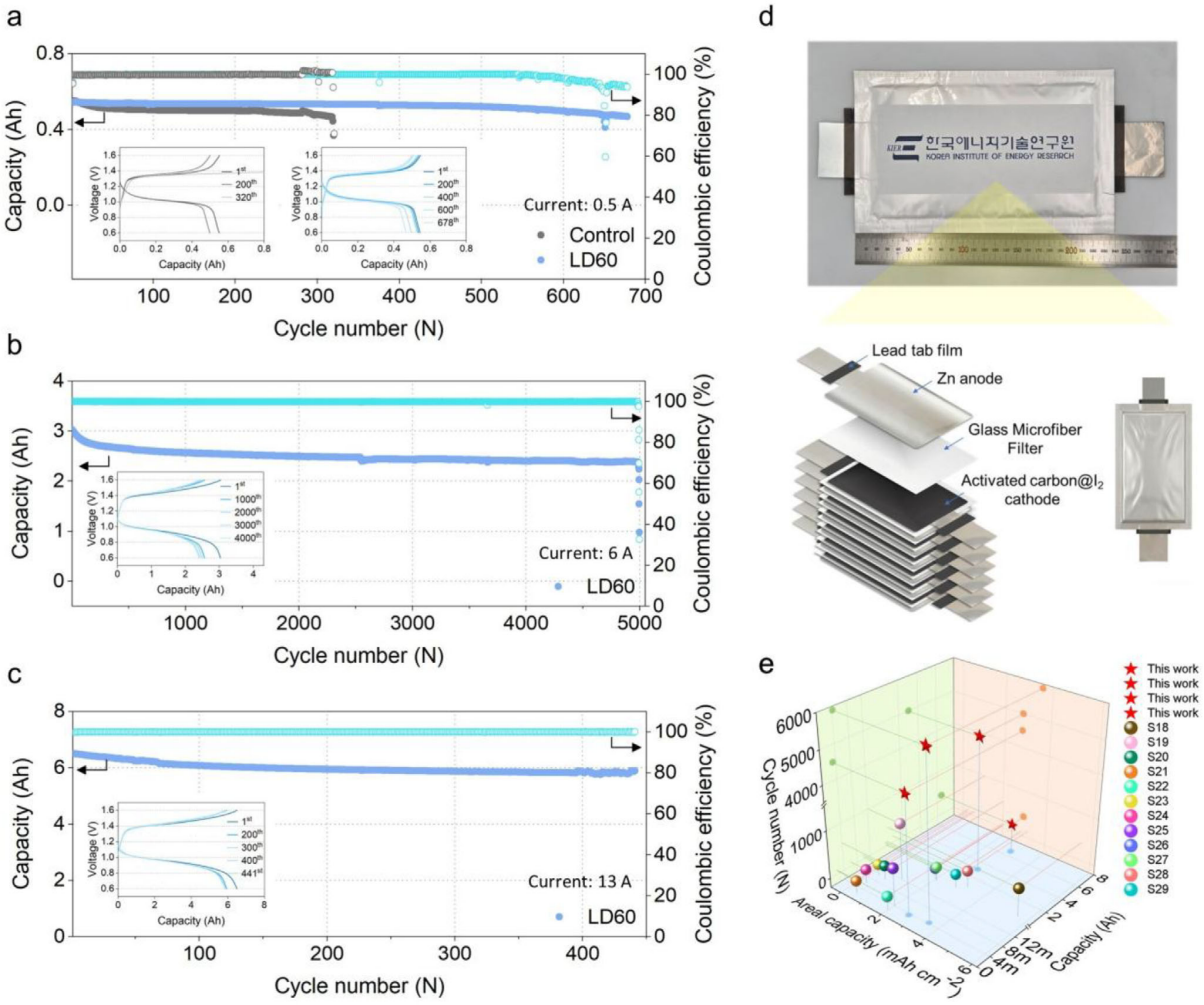
Electrochemical cycling performance of Zn||I_2_ pouch cells with the LD60 electrode at a rate of 1C for (a) 0.5 Ah, and 2C for (b) 3 Ah and (c) 6.5 Ah capacities. (d) Photographs of the stacked cell components used for assembling the 6.5 Ah Zn||I_2_ pouch cell with dimensions of 10 × 18 cm^2^. (e) Comparison of the full‐cell cycling performance of the present study with previously reported studies in terms of cell capacity, areal capacity, and cycle number.

A larger‐format cell with an active area of 10 × 18 cm^2^ exhibited an initial capacity of 3.0 Ah (Figure 7b). This cell demonstrated excellent durability, achieving 81.7% capacity retention over 2400 cycles with a high average Coulombic efficiency of 99.9%. These results demonstrate that the advantages of laser patterning also apply to large‐area, multi‐layer configurations relevant to practical energy storage applications. Crucially, the largest 6.5 Ah cell (Figure 7c) exhibited a stable discharge capacity retention of 90.7% and a high Coulombic efficiency of 99.9% over more than 440 cycles. Figure 7d illustrates the cell components and stacking configuration, along with a photograph of the 6.5 Ah pouch cell with practical dimensions of approximately 10 × 18 cm^2^. As shown in Figure S35, the voltage polarization remained nearly unchanged even as the pouch‐cell capacity increased, demonstrating the excellent scalability of the laser‐patterned Zn anode system. Figure 7e shows a comprehensive 3D benchmarking analysis comparing the full‐cell cycling performance of the present study with previously reported large‐scale Zn||I_2_ pouch cells in terms of total cell capacity, areal capacity, and cycle number (Table S3). This comparison shows that the LD60 electrode configuration delivers the best cycling performance reported for AZIBs, while also achieving the highest cell capacity (>6 Ah), the largest areal capacity (3.6 mAh cm^−2^), and the longest cycle life (5894 cycles), highlighting its strong potential for practical large‐scale energy storage applications.

## 3 Conclusions

We developed a laser‐guided surface patterning strategy that established a resistance gradient to direct epitaxial Zn growth in AZIBs. Periodic micro‐holes selectively removed resistive surface layers, concentrating Zn^2+^ flux and reconstructing the surface texture to favor the stable (002) plane. This architecture facilitated a bottom‐up filling mechanism that effectively suppressed dendrite proliferation and parasitic H_2_ evolution. Optimized LD60 anodes achieved over 5800 cycles in Zn||I_2_ full cells with 67% capacity retention and 99.9% Coulombic efficiency. The practical viability of this approach was validated by a large‐format 6.5 Ah pouch cell (10 × 18 cm^2^), which sustained 441 cycles with 91% capacity retention. This work provides a scalable pathway for engineering 3D architectures to achieve high‐performance, grid‐scale aqueous energy storage.

## 4 Methods

### 4.1 COMSOL Simulations

The simulated cell consisted of an asymmetric Zn||Zn configuration (Figure S9). A series of patterned holes was modeled with dimensions matching the experimental parameters (depths of 20, 60, 100, and 140 µm; inter‐hole spacing of 170 µm). The parameters used in the simulation were listed in Table S1. A phase‐field model coupled with electrochemical kinetics and ion transport was implemented to simulate the morphological evolution of Zn metal during electrodeposition. The spatial distribution of the Zn metal and electrolyte phases was described by the non‐dimensional phase‐field variable ξ, where ξ  =  1 represented the metallic Zn and ξ  =  0 represented the electrolyte phase. The evolution of this phase field was governed by the Cahn–Hilliard equation, which tracks the interface position through the following expression:

$$\;\frac{\partial \xi }{\partial t}=\nabla \cdot \left[M\nabla \left(\frac{\partial f_{bulk}}{\partial \xi }-\kappa \nabla^{2}\xi \right)\right]-\frac{i_{loc}\Omega }{nF}|\nabla \xi |$$

where *M* was the phase‐field mobility, *f_bulk_* was the bulk free energy density (formulated as a double‐well potential), κ was the gradient energy coefficient related to the interfacial energy, Ω was the molar volume of Zn, *n* was the number of electrons transferred (*n*  =  2 for Zn), *F* was the Faraday constant, and *i_loc_* was the local source term representing the electrochemical reaction rate. The transport of ions in the electrolyte was governed by the Nernst–Planck equation, which accounts for the diffusion and migration of Zn^2+^ in the liquid phase. The ionic current density i_*l*_ was expressed as:

$$\;i_{l}=-nFD_{l}\nabla c_{l}-\frac{n^{2}F^{2}D_{l}}{RT}c_{l}\nabla \phi_{l}$$

where *D_l_* was the diffusion coefficient of Zn^2+^, *c_l_* was its concentration, $\phi_{l}$ was the electrolyte potential, *R* was the universal gas constant, and *T* was the absolute temperature. The charge transfer at the Zn/electrolyte interface was modeled using the Butler–Volmer equation, which defines the local current density *i_loc_* as a function of the overpotential η:

$$\;i_{loc}=i_{0}\;\left[\exp\left(\frac{\alpha_{a}nF\eta }{RT}\right)-\exp\left(-\frac{\alpha_{c}nF\eta }{RT}\right)\right]$$

where *i*_0_ was the exchange current density, and α_*a*_ and α_*c*_ were the anodic and cathodic transfer coefficients, respectively. The overpotential was defined as $\eta =\phi_{s}\;-\phi_{l}-E_{eq}$, where $\phi_{s}$ was the electric potential of Zn metal and *E_eq_* was the equilibrium potential. This coupled system of equations allowed for the simultaneous calculation of ion transport, electric field distribution, and the moving boundary of the Zn dendrites during the electrodeposition process. The exchange current density ratio was estimated from the charge‐transfer resistance obtained from the impedance spectra (Figure 4d) by considering the untreated surface and the laser‐exposed hole interiors as regions with distinct exchange current densities (*i*_0_ and *i*_0,hole_, respectively). Approximating the holes as hemi‐ellipsoidal cavities and accounting for their respective surface areas yielded an estimated ratio of approximately five. Based on this estimate, *i*_0,hole_ = 5 *i*_0_ was adopted for the hole‐depth‐dependent simulations.

### 4.2 Materials

Commercial Zn foils (99.95% purity, 200 µm thick) were purchased from Goodfellow and used as the substrate for the anodes. Zinc sulfate heptahydrate (ZnSO_4_·7H_2_O, 99%, Sigma‐Aldrich) and iodine (I_2_, ≥99.8%, Sigma‐Aldrich) were used as the electrolyte salt and the active material, respectively. For the preparation of the dry‐process cathode, two types of activated carbon, MSC30ss and ACS25, were employed. Super P was used as the conductive agent, and polytetrafluoroethylene (PTFE, 100%, Daikin Industries, Ltd) served as the binder. Glass fiber filters (Whatman, GF/C for coin cells and Whatman, GF/D for 3 and 6.5 Ah pouch cells) were utilized as separators. Deionized water was used for all aqueous solutions. All chemical reagents were used as received without further purification.

### 4.3 Preparation of Laser‐Patterned Zn Anodes

To create periodic 3D architectures on the anode surface, commercial Zn foils were patterned using a fiber laser system (20 W/30 W, LPT‐MLCM). Concave micro‐hole patterns were precisely fabricated with a fixed diameter of 60 µm and a center‐to‐center spacing of 170 µm. The micro‐holes were arranged in a uniform 2D array along both the X and Y axes. To investigate the effect of pattern geometry on electrochemical performance, the ablation depth was systematically controlled by varying the number of laser scans (10, 30, 50, and 70 repetitions), resulting in four different depths: 20, 60, 100, and 140 µm; the corresponding samples were denoted as LD20, LD60, LD100, and LD140. For comparison, a laser‐hatched Zn (HC) electrode was prepared by laser irradiation without forming concave micro‐structures, and bare Zn foil was used as a Control electrode. All laser‐processed electrodes were rinsed with ethanol to remove any debris before cell assembly.

### 4.4 Preparation of I_2_‐Impregnated Dry‐Process Cathodes

Cathodes were fabricated using a solvent‐free dry process to ensure structural integrity. First, a carbon blend was prepared by mixing two types of activated carbon, MSC30ss and ACS25, in a 1:1 weight ratio. This blend was then mixed with Super P (conductive agent) and PTFE (binder) in a weight ratio of 9:0.5:0.5. After thorough mixing, the mixture was rolled into a free‐standing film with a uniform thickness using a roll press (Figure S30). The resulting dry‐process electrode was cut to the desired dimensions and attached to a 10 µm primer‐coated titanium current collector (Figure S31). The attachment was performed by applying a pressure of 300 bar for 1 min using a hydraulic press to ensure robust lamination (Figure S32). To incorporate the active material, the as‐prepared dry electrodes were placed in a sealed glass vessel along with solid I_2_ crystals. The vessel was heated in an oven at 80 °C overnight, allowing the I_2_ to impregnate the micropores of the activated carbon through sublimation. The final I_2_ loading was controlled by monitoring the weight difference before and after the impregnation process to achieve an areal capacity ranging from 2.8 to 3.6 mAh cm^−2^.

### 4.5 Cell Assembly

The electrochemical cells were assembled using the prepared laser‐patterned Zn anodes and I_2_‐impregnated cathodes. For the coin‐type cells, CR2032 units were fabricated using a glass fiber separator (Whatman, GF/C) and 2 M ZnSO_4_ aqueous electrolyte. For the pouch‐type cells, three different capacities (0.5, 3, and 6.5 Ah) were designed. The 0.5 Ah pouch cells were assembled using electrodes cut to 6 × 12 cm^2^, with a GF/C separator. For the large‐scale evaluation, 3 and 6.5 Ah pouch cells were constructed using electrodes with dimensions of 10 × 18 cm^2^. To achieve the target capacities, the 3 Ah cells were configured by stacking 6 layers of double‐sided cathodes on primer‐coated current collectors, while the 6.5 Ah cells used 12 layers in the same process. In these high‐capacity pouch cells, a glass fiber separator (Whatman, GF/D) was employed to accommodate the larger electrode area and ensure stable electrolyte wetting. All cells were configured in either symmetric or full‐cell arrangements depending on the experimental requirements. The electrolyte volume was precisely controlled to maintain consistent testing conditions across all cell configurations.

### 4.6 Electrochemical Measurements and Characterization

Electrochemical cycling tests, including galvanostatic charge–discharge (GCD) profiles, were conducted using a battery cycler (WONATech). Electrochemical impedance spectroscopy (EIS) was performed using a Bio‐Logic potentiostat/galvanostat in the frequency range from 1 MHz to 0.1 Hz to monitor the interfacial resistance and ion transport kinetics. To evaluate the hydrogen evolution reaction (HER), linear sweep voltammetry (LSV) and Tafel polarization measurements were carried out in a three‐electrode configuration. To quantify the H_2_ evolution during electrochemical reactions, a displacement method using a hydrometer was employed. To ensure high measurement precision, the internal reservoir of the hydrometer was filled with triple‐distilled water, and all measurements were conducted at a constant temperature to eliminate thermal expansion errors. The volume of generated gas was precisely determined by monitoring the fluid displacement or density changes within the system. In addition, the surface pressure of the pouch cells was monitored in real‐time using a pressure measurement system (Kaitronics, Korea) to analyze the stress evolution during long‐term cycling. The surface and cross‐sectional morphologies of the bare and patterned Zn anodes were examined using field‐emission scanning electron microscopy (FE‐SEM; Regulus 8220, Hitachi). The three‐dimensional surface profiles and ablation depths were analyzed using a confocal optical profiler (PLu Neox, Sensofar). The surface chemical composition and oxidation states were analyzed by X‐ray photoelectron spectroscopy (XPS; Nexsa G2, Thermo Scientific) and time‐of‐flight secondary ion mass spectrometry (TOF‐SIMS; TOF‐SIMS5). The crystallographic orientation and texture, specifically the I_(002)_/I_(101)_ intensity ratio, were evaluated using X‐ray diffraction (XRD; Smart Lab High Resolution, Rigaku) and electron backscatter diffraction (EBSD; TSL Hikari Super). The XRD patterns were recorded in the 2*θ* range of 5°–80° with a scanning speed of 5° min^−1^. In addition, electrolyte wettability was assessed by measuring the static contact angle of a 2 M ZnSO_4_ aqueous electrolyte using a contact angle analyzer.

## Author Contributions

**Suyeon Kim**: methodology, validation, conceptualization. **Dongjae Kim**: visualization, software, data curation. **Youngeun Kim**: validation, methodology. **Chaejeong Kim**: software, formal analysis. **Myunghyun Ryu**: data curation, methodology. **Wooyoung Jin**: data curation, methodology. **Hyeokjin Kwon**: visualization, software. **Kyu‐Nam Jung**: resources, project administration, funding acquisition. **Ki Jae Kim**: validation, methodology, supervision. **Jong‐Won Lee**: supervision, software, writing – review and editing. **Jinhong Lee**: supervision, writing – original draft, writing – review and editing.

## Conflicts of Interest

The authors declare no conflicts of interest.

## Supporting information




**Supporting File 1**: advs78252‐sup‐0001‐SuppMat.docx.

**Supporting File 2**: advs78252‐sup‐0002‐VideoS1.avi.

**Supporting File 3**: advs78252‐sup‐0003‐VideoS2.avi.

**Supporting File 4**: advs78252‐sup‐0004‐VideoS3.avi.

**Supporting File 5**: advs78252‐sup‐0005‐VideoS4.avi.

## Data Availability

The data that support the findings of this study are available on request from the corresponding author. The data are not publicly available due to privacy or ethical restrictions.
