# Supplementary material for: Controlling Epitaxial Zn Growth via Guided Laser Patterning toward High‐Performance 6Ah Aqueous Zn||I2 Batteries
Source: Adv Sci (Weinh). 2026 Oct 15:e78252. Online ahead of print. doi: 10.1002/advs.78252 (PMC13672250; doi:10.1002/advs.78252)
Supplement: Supplementary file 1 [file ADVS-9999-e78252-s004.docx]

Supporting Information

**Controlling epitaxial Zn growth via guided laser patterning toward high-performance 6Ah aqueous Zn||I_2_ batteries**

Suyeon Kim, ^‡^ Dongjae Kim, ^‡^ Youngeun Kim, Chaejeong Kim, Myunghyun Ryu, Wooyoung Jin, Hyeokjin Kwon, Kyu-Nam Jung, Ki Jae Kim,* Jong-Won Lee,* and Jinhong Lee*

^‡^ Equally contributed to this work.

* Corresponding Author

**Figure S1**. Cross-sectional and backside SEM images of the Control and LD60 Zn electrodes.

**Figure S2**. Water contact angle measurements of a 2 M ZnSO_4_ aqueous electrolyte on HC and LD20.

**Figure S3**. Comparison of the water contact angle for different patterned hole depths.

Capillary suction in each cylindrical hole provides the dominant driving force for infiltration, with $P_{c}=\frac{2\gamma\cos\theta}{r_{c}}$. For an aqueous electrolyte $\gamma(surface tension)\approx0.07\text{ N/m}$ and $r_{c}\approx30\text{ }\mu\text{m}$, yielding $P_{c}$ values of 1.44, 3.91, 4.12, and −3.06 kPa at a depth of 20 (72°), 60 (33°), 100 (28°), and 140 μm (131°), respectively. Positive capillary pressures indicate spontaneous electrolyte infiltration into the concave holes, whereas the negative pressure observed at a contact angle of 131° signifies suppressed wetting and capillary resistance.

**Figure S4**. Comparison of the initial Zn deposition overpotentials at (a) 5 mA cm^−2^ and 1 mAh cm^−2^, (b) 5 mA cm^−2^ and 3 mAh cm^−2^, and (c) 10 mA cm^−2^ and 2 mAh cm^−2^, demonstrating that laser treatment effectively removed the resistive surface layer.

**Figure S5**. EIS spectrum of symmetric cells with the Control and HC electrodes, measured after 6 h of aging.

**Figure S6**. Tafel plots of symmetric cells assembled with Control and HC Zn electrodes for determining their exchange currents.

**Figure S7**. XRD patterns of pristine bare Zn, HC, and LD60.

**Figure S8**. SEM images of Zn deposition behavior at a current density of 5 mA cm^−2^. Zn stripping was performed on (a) bare Zn at an areal capacity of 5 mAh cm^−2^ and on a laser-patterned Zn anode with a pattern depth: 60 μm (LD60) at areal capacities of (b) 1, (c) 3, (d) 5, and (e) 10 mAh cm^−2^.

**Figure S9**. The model geometry and activated boundary conditions for the phase-field simulations.

**Figure S10**. Phase-field simulation results of patterned Zn with *i*_0,hole_ = 3 × *i*_0_.

**Figure S11**. Phase-field simulation results of patterned Zn with *i*_0,hole_ = 0.5 × *i*_0_.

**Figure S12**. Phase-field simulation results of patterned Zn with an average hole depth of 20 μm (LD20).

**Figure S13**. Phase-field simulation results of patterned Zn with an average hole depth of 100 μm (LD100).

**Figure S14**. Comparison of Zn||Zn symmetric cell performance reported in the literature with the present system in terms of cycle time, current density, and areal capacity.

**Figure S15**. EIS spectrum of Zn||Zn symmetric cells after 50, 100, and 300 cycles for the LD60 electrode.

**Figure S16**. Surface SEM and confocal microscopy images of the LD60 Zn electrode before and after 300 cycles (Zn symmetric cell 10 mA cm^-2^, 2 mAh cm^-2^).

**Figure S17**. 3D confocal images of the Control, HC, LD20, LD60, LD100, and LD140 electrodes in the pristine state and after the 50th and 100th cycles in Zn||Zn symmetric cells.

**Figure S18**. SEM images of the Control, HC, LD20, LD60, LD100, and LD140 electrodes after 50 cycles in Zn||Zn symmetric cells, showing the evolution of the (101) and (002) lattice-associated deposition morphologies.

**Figure S19**. 3D confocal images and corresponding line-scan profiles of the Control and LD60 electrodes after 50 cycles in Zn||Zn symmetric cells.

**Figure S20**. Analysis of H_2_ evolution behavior in a 1 × 1 cm^2^ beaker cell using Zn||Zn symmetric cells with the Control and LD60 electrodes at 10 mA cm^−2^ and 2 mAh cm^−2^ before cycling and after 50 cycles.

**Figure S21**. Comparison of pouch-cell thickness change and H_2_ evolution volume during storage for the Control and LD60 electrodes.

**Figure S22**. EIS spectra of Zn||Zn symmetric cells measured after aging for 0, 12, 24, 36, and 48 h.

**Figure S23**. Schematic of Zn^2+^ diffusion. Extensive lateral (2D) diffusion on bare Zn leads to non-uniform deposition, whereas 3D diffusion on LD60 promotes a more uniform ion distribution and homogeneous plating.

**Figure S24**. (a) SEM images and (b) XRD patterns of the Control and LD60 Zn electrodes after 500 cycles in Zn||I_2_ cells.

**Figure S25**. SEM images and corresponding EDS elemental maps of (a) Control and (b) LD60 after 500 cycles in Zn||I_2_ cells.

**Figure S26**. SEM images of Control and LD60 Zn electrodes after immersion for 1, 3, 5, and 7 days in 2 M ZnSO_4_ containing 0.1 M I_3_⁻ to assess surface degradation under polyiodide exposure.

**Figure S27**. EIS analysis (1 MHz~0.1 Hz) of Zn||Zn symmetric cells after storage for 1, 3, 5, and 7 days without cycling in 2 M ZnSO_4_ containing 0.1 M I_3_⁻.

**Figure S28**. Voltage profiles of (a) Control and (b) LD60 Zn||I_2_ cells after resting at open circuit for 12, 24, and 48 h at 25 °C following a full charge. (c) Corresponding capacity retention as a function of rest time.

**Figure S29**. Electrochemical cycling performance of Zn||I_2_ coin-type cells operated at a rate of 10C (28 mA cm^−2^) using Control, HC, LD20, LD60, LD100, and LD140 electrodes.

**Figure S30**. Solvent-free dry-process electrode fabrication. (a) Mixed electrode blend. (b) Continuous formation of the free-standing electrode film via a roll-press.

**Figure S31**. Photograph of the primer-coated titanium foils (10 × 18 cm^2^) used as current collectors, showing both the precisely cut sheets and bulk foil in roll form.

**Figure S32**. Detailed lamination process of the dry-process electrode onto the primer-coated titanium current collector. (a) Placement of the free-standing electrode film and titanium foil in a hydraulic press. (b, c) Photographs of the laminated electrode after applying a pressure of 300 bar for 1 min, demonstrating successful adhesion and structural integrity. (Note: These images are representative examples for illustrating the fabrication procedure.)

**Figure S33**. Photograph of the failed 0.5 Ah Zn||I_2_ pouch cell employing the Control electrode.

**Figure S34**. Comparison of the Zn metal surface morphology of the Control and LD60 electrodes in 0.5 Ah Zn||I_2_ cells after cell failure.

**Figure S35**. Voltage profiles of Zn||I_2_ pouch cells with capacities of (a) 0.5, (b) 3.0, and (c) 6.5 Ah.

**Table S1**. Input parameters and values for COMSOL simulations.

| **Parameter** | **Value** | **Unit** |  |
| --- | --- | --- | --- |
| Diffusion coefficient in electrolyte (*D*_l_) | 0.328 × 10^−9^ | m^2^ s^−1^ | [1] |
| Initial concentration in electrolyte (*c*_0_) | 2000 | mol m^−3^ | Experimental |
| Temperature | 298.15 | K | - |
| Exchange current density (*i*_0_) | 20 | A m^−2^ | Assumed |
| Symmetric factor (*α*) | 0.5 | - | [2] |
| Number of electrons transferred | 2 | - | [3] |
| Surface tension coefficient (*σ*) | 1 | J m^−2^ | Assumed |
| Activate site diameter (bare metal) | 10 | μm | - |

**Table S2.** Comparison of the Zn||Zn symmetric cell performance of this work and recent studies.

| **Zn anodes** | **Current density**  **[mA cm^−2^]** | **Areal capacity**  **[mAh cm^−2^]** | **Cycle time**  **[h]** | **Ref.** |
| --- | --- | --- | --- | --- |
| Zn-LIPSS | 5 | 1 | 800 | [4] |
| Fs–Zn | 10 | 1 | 650 | [5] |
| Fs–Zn | 10 | 5 | 155 | [5] |
| LLP@Treated Zn | 2 | 1 | 746 | [6] |
| LLP@Treated Zn | 1 | 1 | 1200 | [6] |
| LT-Zn@ZnO | 2 | 1 | 2500 | [6] |
| LLP@ZF | 0.5 | 0.5 | 1100 | [7] |
| LLP@ZF | 1 | 0.5 | 440 | [7] |
| LLP@ZF | 2 | 1 | 400 | [7] |
| 3D-ULPT-Zn | 2 | 1 | 2530 | [8] |
| ZIF-8@Zn | 2 | 2 | 700 | [9] |
| Zn nanosheets | 0.2 | 0.1 | 850 | [10] |
| Nafion-Zn-X | 1 | 10 | 1000 | [11] |
| Kaolin@Zn | 4.4 | 1.1 | 800 | [12] |
| ZnSe@Zn | 1 | 1 | 1530 | [13] |
| Alucone@Zn | 3 | 1 | 780 | [14] |
| Al_2_O_3_@Zn | 1 | 1 | 500 | [15] |
| Indium-based layer | 4 | 1 | 400 | [16] |
| TiO_2_@Zn | 1 | 1 | 150 | [17] |
| LD60 | 5 | 1 | 1114 | **This**  **work** |
| LD60 | 5 | 3 | 310 |  |
| LD60 | 10 | 2 | 388 |  |

**Table S3.** Comparison of the full cell performance of this work and recent publications.

| **Designation** | **Cell capacity**  **[mAh]** | **Areal capacity**  **[mAh cm^−2^]** | **Cycle number**  **[N]** | **Ref.** |
| --- | --- | --- | --- | --- |
| P-G@Zn | 400 | 5.6 | 400 | [18] |
| CaMnO-140 | 12.15 | 1.08 | 1000 | [19] |
| X=4 | 9.8 | 0.612 | 100 | [20] |
| MS-8 | 1.92 | 0.5 | 90 | [21] |
| 1+1+0.2M HAc | 2 | 2 | 100 | [22] |
| PTM | 10 | 0.25 | 40 | [23] |
| MnO_2_@AEPA | 6 | 0.3 | 110 | [24] |
| HP-Mn_2_O_3_-YSMSs@G film | 4 | 2 | 600 | [25] |
| BPE | 100 | 2 | 50 | [26] |
| DME40 | 62.4 | 2.08 | 100 | [27] |
| Zn@ZnF_2_ | 850 | 3.2 | 160 | [28] |
| ZnSO_4_ with hydrophobic CDs | 261 | 2.9 | 100 | [29] |
| LD60 | 3.2 | 2.8 | 4500 | **This**  **work** |
| LD60 | 4.1 | 3.6 | 5894 |  |
| LD60 | 3000 | 2.8 | 4996 |  |
| LD60 | 6500 | 3 | 441 |  |

**Video 1.** Animation of the phase-field simulation on un-patterned Zn (Control).

**Video 2.** Animation of the phase-field simulation on patterned Zn with *i*_0,hole_ = 0.5 *i*_0_.

**Video 3.** Animation of the phase-field simulation on patterned Zn with 60 μm depth holes (LD60).

**Video 4.** Animation of the phase-field simulation on patterned Zn with 140 μm depth holes (LD140).

1.	J. G. Albright, D. G. Miller, “Mutual diffusion coefficients of aqueous ZnSO_4_ at 25℃,” *Journal of Solution Chemistry* 4 (1975): 809-816, https://doi.org/10.1007/bf00650537.

2.	E. Guerra, G. H. Kelsall, M. Bestetti, D. Dreisinger, K. Wong, K. A. R. Mitchell, D. Bizzotto, “Use of underpotential deposition for evaluation of overpotential deposition kinetics of reactive metals,” *Journal of The Electrochemical Society* 151 (2004): E1-E6, https://doi.org/10.1149/1.1631282.

3.	I. Epelboin, M. Ksouri, R. Wiart, “On a model for the electrocrystallization of zinc involving an autocatalytic step,” *Journal of The Electrochemical Society* 122 (1975): 1206-1214, https://doi.org/10.1149/1.2134426.

4.	Y. Liu, J. Xie, Y. Ding, J. Xu, D. Huang, Y. Wang, S. Chen, Q. Hu, L. Xu, L. Yang, “Dual‐Functional Layer Engineering Unlocking Dendrite‐Free and High‐Performance Zinc Metal Anodes,” *Advanced Functional Materials* 35 (2025): 2424526, https://doi.org/10.1002/adfm.202424526.

5.	S. Lian, Z. Cai, M. Yan, C. Sun, N. Chai, B. Zhang, K. Yu, M. Xu, J. Zhu, X. Pan, Y. Dai, J. Huang, B. Mai, L. Qin, W. Shi, Q. Xin, X. Chen, K. Fu, Q. An, Q. Yu, L. Zhou, W. Luo, K. Zhao, X. Wang, L. Mai, “Ultra‐high proportion of grain boundaries in zinc metal anode spontaneously inhibiting dendrites growth,” *Angewandte Chemie* 136 (2024): e202406292, https://doi.org/10.1002/ange.202406292.

6.	Z. Na, H. Qi, S. Li, Y. Wu, Q. Wang, G. Huang, “Stable zinc metal anode by nanosecond pulsed laser enabled gradient design,” *ACS Energy Letters* 8 (2023): 3297-3306, https://doi.org/10.1021/acsenergylett.3c01206.

7.	Z. Huang, H. Li, Z. Yang, H. Wang, J. Ding, L. Xu, Y. Tian, D. Mitlin, J. Ding, W. Hu, “Nanosecond laser lithography enables concave-convex zinc metal battery anodes with ultrahigh areal capacity,” *Energy Storage Materials* 51 (2022): 273-285, https://doi.org/10.1016/j.ensm.2022.06.054.

8.	Y. Liu, Y. Ding, Z. Liu, X. Li, S. Tian, L. Fan, J. Xie, L. Xu, J. Lee, J. Li, L. Yang “Ultrafast laser one-step construction of 3D micro-/nanostructures achieving high-performance zinc metal anodes,” *PhotoniX* 5 (2024): 6, https://doi.org/10.1186/s43074-024-00122-x.

9.	X. Liu, F. Yang, W. Xu, Y. Zeng, J. He, X. Lu, “Zeolitic imidazolate frameworks as Zn^2+^ modulation layers to enable dendrite‐free Zn anodes,” *Advanced Science* 7 (2020): 2002173, https://doi.org/10.1002/advs.202002173.

10.	C. Wu, H. Tan, W. Huang, W. Li, K. N. Dinh, C. Yan, W. Wei, L. Chen, Q. Yan, “A new scalable preparation of metal nanosheets: potential applications for aqueous Zn‐ion batteries anode,” *Advanced Functional Materials* 30 (2020): 2003187, https://doi.org/10.1002/adfm.202003187.

11.	Y. Cui, Q. Zhao, X. Wu, X. Chen, J. Yang, Y. Wang, R. Qin, S. Ding, Y. Song, J. Wu, “An interface‐bridged organic–inorganic layer that suppresses dendrite formation and side reactions for ultra‐long‐life aqueous zinc metal anodes,” *Angewandte Chemie International Edition* 59 (2020): 16594-16601, https://doi.org/10.1002/anie.202005472.

12.	C. Deng, X. Xie, J. Han, Y. Tang, J. Gao, C. Liu, X. Shi, J. Zhou, S. Liang, “A sieve‐functional and uniform‐porous kaolin layer toward stable zinc metal anode,” *Advanced Functional Materials* 30 (2020): 2000599, https://doi.org/10.1002/adfm.202000599.

13.	X. Yang, C. Li, Z. Sun, S. Yang, Z. Shi, R. Huang, B. Liu, S. Li, Y. Wu, M. Wang, “Interfacial manipulation via in situ grown ZnSe cultivator toward highly reversible Zn metal anodes,” *Advanced Materials* 33 (2021): 2105951, https://doi.org/10.1002/adma.202105951.

14.	H. He, J. Liu, “Suppressing Zn dendrite growth by molecular layer deposition to enable long-life and deeply rechargeable aqueous Zn anodes,” *Journal of Materials Chemistry A* 8 (2020): 22100-22110, https://doi.org/10.1039/D0TA07232J.

15.	H. He, H. Tong, X. Song, X. Song, J. Liu, “Highly stable Zn metal anodes enabled by atomic layer deposited Al_2_O_3_ coating for aqueous zinc-ion batteries,” *Journal of Materials Chemistry A* 8 (2020): 7836-7846, https://doi.org/10.1039/D0TA00748J.

16.	K. Hu, X. Guan, R. Lv, G. Li, Z. Hu, L. Ren, A. Wang, X. Liu, J. Luo, “Stabilizing zinc metal anodes by artificial solid electrolyte interphase through a surface ion-exchanging strategy,” *Chemical Engineering Journal* 396 (2020): 125363, https://doi.org/10.1016/j.cej.2020.125363.

17.	K. Zhao, C. Wang, Y. Yu, M. Yan, Q. Wei, P. He, Y. Dong, Z. Zhang, X. Wang, L. Mai, “Ultrathin surface coating enables stabilized zinc metal anode,” *Advanced Materials Interfaces* 5 (2018): 1800848, https://doi.org/10.1002/admi.201800848.

18.	L. Yang, Y. Shen, X. Long, Q. Ma, Z. Ruan, N. Xu, K. Li, L. Jiao, Y. Kong, J. Li, “Engineering an ion-pumping solid electrolyte interphase for ultra-stable aqueous zinc-ion batteries under deep discharge conditions,” *Energy & Environmental Science* 18.18 (2025): 8667-8678, https://doi.org/10.1039/D5EE01408E.

19.	T. Wang, J. Jin, X. Zhao, X. Qu, L. Jiao, Y. Liu, “Unraveling the Anionic Redox Chemistry in Aqueous Zinc‐MnO_2_ Batteries,” *Angewandte Chemie* 136 (2024): e202412057, https://doi.org/10.1002/ange.202412057.

20.	Y. Hu, Z. Liu, L. Li, S. Guo, X. Xie, Z. Luo, G. Fang, S. Liang, “Reconstructing interfacial manganese deposition for durable aqueous zinc–manganese batteries,” *National Science Review* 10 (2023): nwad220, https://doi.org/10.1093/nsr/nwad220.

21.	M. Chen, W. Liu, D. Ren, Y. An, C. Shu, S. Zhang, W. Liang, J. Sun, F. Kang, F. Jiang, “Proton Self‐Limiting Effect of Solid Acids Boosts Electrochemical Performance of Zinc‐ion Batteries,” *Advanced Functional Materials* 34 (2024): 2404983, https://doi.org/10.1002/adfm.202404983.

22.	Z. Liu, Y. Yang, S. Liang, B. Lu, J. Zhou, “pH‐buffer contained electrolyte for self‐adjusted cathode‐free Zn–MnO_2_ batteries with coexistence of dual mechanisms,” *Small Structures* 2 (2021): 2100119, https://doi.org/10.1002/sstr.202100119.

23.	X. Huang, X. Liu, H. Li, Q. Zhao, T. Ma, “Revealing the real charge carrier in aqueous zinc batteries based on polythiophene/manganese dioxide cathode,” *Small Structures* 4 (2023): 2200221, https://doi.org/10.1002/sstr.202200221.

24.	X. Xiao, L. Zhang, W. Xin, M. Yang, Y. Geng, M. Niu, H. Zhang, Z. Zhu, “Self‐assembled layer of organic phosphonic acid enables highly stable MnO_2_ cathode for aqueous zinc batteries,” *Small* 20 (2024): 2309271, https://doi.org/10.1002/smll.202309271.

25.	H. Luo, B. Wang, J. Jian, F. Wu, L. Peng, D. Wang, “Stress-release design for high-capacity and long-time lifespan aqueous zinc-ion batteries,” *Materials Today Energy* 21 (2021): 100799, https://doi.org/10.1016/j.mtener.2021.100799.

26.	J. Zhu, M. Yang, Y. Hu, M. Yao, J. Chen, Z. Niu, “The construction of binary phase electrolyte interface for highly stable zinc anodes,” *Advanced Materials* 36 (2024): 2304426, https://doi.org/10.1002/adma.202304426.

27.	G. Ma, L. Miao, Y. Dong, W. Yuan, X. Nie, S. Di, Y. Wang, L. Wang, N. Zhang, “Reshaping the electrolyte structure and interface chemistry for stable aqueous zinc batteries,” *Energy Storage Materials* 47 (2022): 203-210, https://doi.org/10.1016/j.ensm.2022.02.019.

28.	L. Ma, Q. Li, Y. Ying, F. Ma, S. Chen, Y. Li, H. Huang, C. Zhi, “Toward practical high‐areal‐capacity aqueous zinc‐metal batteries: quantifying hydrogen evolution and a solid‐ion conductor for stable zinc anodes,” *Advanced Materials* 33 (2021): 2007406, https://doi.org/10.1002/adma.202007406.

29.	H. Yang, K. Zhu, W. Xie, W. Yang, “Enhancing zinc anode reversibility through dynamic interface engineering with monolayer hydrophobic carbon dots,” *ACS Nano* 19 (2025): 1433-1446, https://doi.org/10.1021/acsnano.4c14244.
